# 6-Meth­oxy-2-methyl-1-phenyl-1*H*-indole-3-carbonitrile

**DOI:** 10.1107/S1600536811038724

**Published:** 2011-09-30

**Authors:** Qiao Yan, Xiuxiang Qi

**Affiliations:** a School of Pharmaceutical Science and Technology, Tianjin University, Tianjin 300072, People’s Republic of China

## Abstract

In the title compound, C_17_H_14_N_2_O, the dihedral angle between the indole ring system and the phenyl ring is 64.48 (7)°. The crystal packing features weak C—H⋯π inter­actions.

## Related literature

For the synthesis of the title compound, see: Du *et al.* (2006 ▶). For its precursor, see: Jin *et al.* (2009 ▶). For related structures, see: Yang *et al.* (2011 ▶); Yan & Qi (2011*a* ▶,*b* ▶).
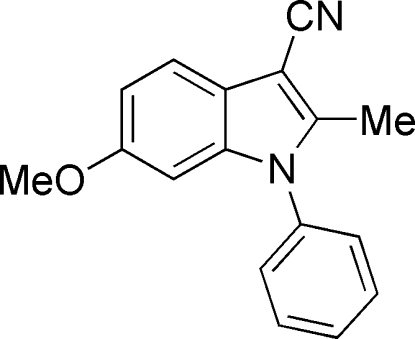


## Experimental

### 

#### Crystal data

C_17_H_14_N_2_O
*M* *_r_* = 262.30
Triclinic, 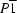

*a* = 6.3699 (7) Å
*b* = 10.6084 (10) Å
*c* = 10.8139 (11) Å
α = 70.059 (10)°
β = 79.455 (13)°
γ = 78.869 (12)°
*V* = 668.55 (12) Å^3^
*Z* = 2
Mo *K*α radiation
μ = 0.08 mm^−1^
*T* = 113 K
0.24 × 0.20 × 0.20 mm

#### Data collection

Rigaku Saturn724 CCD diffractometer
Absorption correction: multi-scan (*CrystalClear-SM Expert*; Rigaku, 2009 ▶) *T*_min_ = 0.981, *T*_max_ = 0.984
7078 measured reflections
3157 independent reflections
1404 reflections with *I* > 2σ(*I*)
*R*_int_ = 0.052

#### Refinement

*R*[*F* ^2^ > 2σ(*F* ^2^)] = 0.041
*wR*(*F* ^2^) = 0.095
*S* = 0.89
3157 reflections
184 parameters
H-atom parameters constrained
Δρ_max_ = 0.36 e Å^−3^
Δρ_min_ = −0.25 e Å^−3^

### 

Data collection: *CrystalClear-SM Expert* (Rigaku, 2009 ▶); cell refinement: *CrystalClear-SM Expert*; data reduction: *CrystalClear-SM Expert*; program(s) used to solve structure: *SHELXS97* (Sheldrick, 2008 ▶); program(s) used to refine structure: *SHELXL97* (Sheldrick, 2008 ▶); molecular graphics: *CrystalStructure* (Rigaku, 2009 ▶); software used to prepare material for publication: *CrystalStructure*.

## Supplementary Material

Crystal structure: contains datablock(s) global, I. DOI: 10.1107/S1600536811038724/bt5647sup1.cif

Structure factors: contains datablock(s) I. DOI: 10.1107/S1600536811038724/bt5647Isup2.hkl

Supplementary material file. DOI: 10.1107/S1600536811038724/bt5647Isup3.cml

Additional supplementary materials:  crystallographic information; 3D view; checkCIF report

## Figures and Tables

**Table 1:** Hydrogen-bond geometry (Å, °) *Cg*1, *Cg*2 and *Cg*3 are the centroids of the N1/C1/C6–C8, C1–C6 and C12–C17 rings, respectively.

*D*—H⋯*A*	*D*—H	H⋯*A*	*D*⋯*A*	*D*—H⋯*A*
C9—H9*B*⋯*Cg*2^i^	0.98	2.78	3.701 (2)	156
C10—H10*B*⋯*Cg*3^ii^	0.98	2.65	3.516 (2)	148
C10—H10*C*⋯*Cg*1^iii^	0.98	2.73	3.509 (2)	137

Symmetry codes: (i) 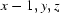; (ii) 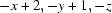; (iii) 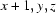.

**Fig. 1.**
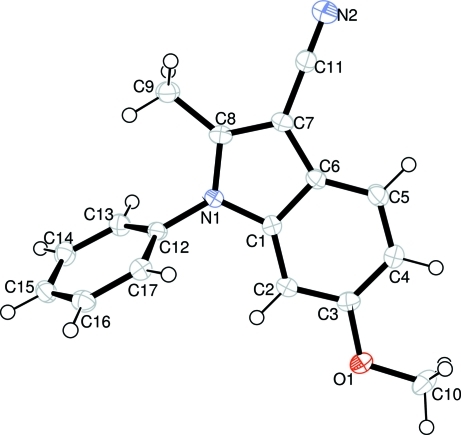
The molecular structure of molecule one of (I) with the atom-numbering scheme and 50% probability displacement ellipsoids.

## supplementary crystallographic information

### Comment

In our continuous investigation about indole derivatives, herein, we report the title compound (I). In the molecular structure (Fig. 1), the indole ring is almost planar with a dihedral angle of 1.37 (10)° between its pyrrole ring and fused benzene ring, greater than those in 1-(2-chlorophenyl)- 6-fluoro-2-methyl-1*H*-indole-3-carbonitrile [0.85 (6)°] (Yang *et al.*, 2011) and 1-(4-bromophenyl)-2-methyl-1*H*-indole-3-carbonitrile [0.95 (16)°] (Yan & Qi, 2011*b*), but less than that [2.66 (6)°] of our previously reported 1-(4-methoxyphenyl)-2-methyl-1*H*-indole-3-carbonitrile (Yan & Qi, 2011*a*).

The indole ring forms an angle of 64.48 (7)° with the phenyl ring, being between those [58.41 (4)° & 58.85 (11) °] reported by our group (Yan & Qi, 2011*a*,*b*), and that [80.91 (5)°] reported by Yang *et al.* (2011).

In the crystal packing, weak C—H···π interaction were observed, establishing the packing (Table 1).

### Experimental

The title compound was prepared according to the method of the literature (Du, *et al.*, 2006). Colourless prisms   were grown from a mixture of ethyl acetate and petroleum ether.

### Refinement

All H atoms were positioned geometrically (C—H = 0.95 and 0.98 Å)and refined as riding with *U*_iso_(H) = 1.2*U*_eq_(CH) or 1.5*U*_eq_(CH_3_).

### Crystal data

**Table:**

C_17_H_14_N_2_O	*Z* = 2
*M**_r_* = 262.30	*F*(000) = 276
Triclinic, *P*1	*D*_x_ = 1.303 Mg m^−^^3^
Hall symbol: -P 1	Mo *K*α radiation, λ = 0.71073 Å
*a* = 6.3699 (7) Å	Cell parameters from 2377 reflections
*b* = 10.6084 (10) Å	θ = 2.0–28.1°
*c* = 10.8139 (11) Å	µ = 0.08 mm^−^^1^
α = 70.059 (10)°	*T* = 113 K
β = 79.455 (13)°	Prism, colorless
γ = 78.869 (12)°	0.24 × 0.20 × 0.20 mm
*V* = 668.55 (12) Å^3^	

### Data collection

**Table:**

Rigaku Saturn724 CCD diffractometer	3157 independent reflections
Radiation source: fine-focus sealed tube	1404 reflections with *I* > 2σ(*I*)
graphite	*R*_int_ = 0.052
Detector resolution: 14.22 pixels mm^-1^	θ_max_ = 28.0°, θ_min_ = 2.0°
ω and φ scans	*h* = −8→8
Absorption correction: multi-scan (*CrystalClear*; Rigaku, 2009)	*k* = −13→13
*T*_min_ = 0.981, *T*_max_ = 0.984	*l* = −14→14
7078 measured reflections	

### Refinement

**Table:**

Refinement on *F*^2^	Secondary atom site location: difference Fourier map
Least-squares matrix: full	Hydrogen site location: inferred from neighbouring sites
*R*[*F*^2^ > 2σ(*F*^2^)] = 0.041	H-atom parameters constrained
*wR*(*F*^2^) = 0.095	*w* = 1/[σ^2^(*F*_o_^2^) + (0.0296*P*)^2^] where *P* = (*F*_o_^2^ + 2*F*_c_^2^)/3
*S* = 0.89	(Δ/σ)_max_ < 0.001
3157 reflections	Δρ_max_ = 0.36 e Å^−^^3^
184 parameters	Δρ_min_ = −0.25 e Å^−^^3^
0 restraints	Extinction correction: *SHELXL*, Fc^*^=kFc[1+0.001xFc^2^λ^3^/sin(2θ)]^-1/4^
Primary atom site location: structure-invariant direct methods	Extinction coefficient: 0.048 (4)

### Special details

**Table:**

Geometry. All e.s.d.'s (except the e.s.d. in the dihedral angle between two l.s. planes) are estimated using the full covariance matrix. The cell e.s.d.'s are taken into account individually in the estimation of e.s.d.'s in distances, angles and torsion angles; correlations between e.s.d.'s in cell parameters are only used when they are defined by crystal symmetry. An approximate (isotropic) treatment of cell e.s.d.'s is used for estimating e.s.d.'s involving l.s. planes.
Refinement. Refinement of *F*^2^ against ALL reflections. The weighted *R*-factor *wR* and goodness of fit *S* are based on *F*^2^, conventional *R*-factors *R* are based on *F*, with *F* set to zero for negative *F*^2^. The threshold expression of *F*^2^ > σ(*F*^2^) is used only for calculating *R*-factors(gt) *etc*. and is not relevant to the choice of reflections for refinement. *R*-factors based on *F*^2^ are statistically about twice as large as those based on *F*, and *R*- factors based on ALL data will be even larger.

### Fractional atomic coordinates and isotropic or equivalent isotropic displacement parameters (Å^2^)

**Table:**

	*x*	*y*	*z*	*U*_iso_*/*U*_eq_	
O1	1.04602 (18)	0.50144 (11)	0.16822 (10)	0.0270 (3)	
N1	0.4880 (2)	0.22413 (13)	0.23013 (11)	0.0196 (3)	
N2	0.1580 (2)	0.13570 (16)	0.67436 (13)	0.0360 (4)	
C1	0.6199 (3)	0.29364 (16)	0.26611 (14)	0.0191 (4)	
C2	0.7805 (3)	0.36887 (16)	0.18802 (15)	0.0211 (4)	
H2	0.8173	0.3779	0.0965	0.025*	
C3	0.8845 (3)	0.42998 (17)	0.24916 (15)	0.0226 (4)	
C4	0.8278 (3)	0.41970 (18)	0.38350 (15)	0.0260 (4)	
H4	0.9008	0.4634	0.4228	0.031*	
C5	0.6652 (3)	0.34576 (17)	0.45878 (15)	0.0257 (4)	
H5	0.6261	0.3392	0.5496	0.031*	
C6	0.5590 (3)	0.28104 (17)	0.40116 (14)	0.0203 (4)	
C7	0.3836 (3)	0.20116 (17)	0.44466 (14)	0.0220 (4)	
C8	0.3453 (3)	0.16698 (17)	0.33986 (15)	0.0217 (4)	
C9	0.1853 (3)	0.08298 (18)	0.33621 (15)	0.0264 (4)	
H9A	0.2120	0.0657	0.2508	0.040*	
H9B	0.0392	0.1314	0.3472	0.040*	
H9C	0.1996	−0.0033	0.4082	0.040*	
C10	1.1759 (3)	0.55522 (18)	0.22829 (15)	0.0303 (5)	
H10A	1.0862	0.6254	0.2622	0.045*	
H10B	1.2925	0.5947	0.1620	0.045*	
H10C	1.2377	0.4823	0.3016	0.045*	
C11	0.2601 (3)	0.16396 (18)	0.57217 (16)	0.0261 (4)	
C12	0.5114 (3)	0.20522 (17)	0.10210 (14)	0.0194 (4)	
C13	0.3439 (3)	0.25740 (17)	0.02478 (15)	0.0238 (4)	
H13	0.2171	0.3088	0.0539	0.029*	
C14	0.3643 (3)	0.23346 (17)	−0.09557 (15)	0.0266 (4)	
H14	0.2488	0.2655	−0.1480	0.032*	
C15	0.5530 (3)	0.16287 (17)	−0.13911 (15)	0.0251 (4)	
H15	0.5666	0.1472	−0.2218	0.030*	
C16	0.7218 (3)	0.11496 (17)	−0.06358 (15)	0.0253 (4)	
H16	0.8522	0.0684	−0.0952	0.030*	
C17	0.7001 (3)	0.13519 (17)	0.05889 (15)	0.0227 (4)	
H17	0.8142	0.1011	0.1123	0.027*	

### Atomic displacement parameters (Å^2^)

**Table:**

	*U*^11^	*U*^22^	*U*^33^	*U*^12^	*U*^13^	*U*^23^
O1	0.0275 (8)	0.0277 (8)	0.0277 (6)	−0.0125 (6)	0.0001 (5)	−0.0083 (6)
N1	0.0208 (8)	0.0226 (9)	0.0161 (7)	−0.0061 (7)	0.0003 (6)	−0.0068 (6)
N2	0.0381 (11)	0.0441 (11)	0.0254 (8)	−0.0143 (8)	0.0003 (7)	−0.0082 (8)
C1	0.0194 (10)	0.0185 (9)	0.0192 (8)	−0.0011 (8)	−0.0035 (7)	−0.0061 (7)
C2	0.0219 (10)	0.0211 (10)	0.0181 (8)	−0.0020 (8)	−0.0005 (7)	−0.0052 (7)
C3	0.0215 (10)	0.0199 (10)	0.0243 (9)	−0.0040 (8)	0.0002 (8)	−0.0052 (7)
C4	0.0269 (11)	0.0283 (11)	0.0262 (9)	−0.0073 (9)	−0.0047 (8)	−0.0105 (8)
C5	0.0316 (11)	0.0274 (11)	0.0179 (8)	−0.0061 (9)	−0.0034 (8)	−0.0058 (8)
C6	0.0207 (10)	0.0218 (10)	0.0169 (8)	−0.0015 (8)	−0.0025 (7)	−0.0049 (7)
C7	0.0239 (11)	0.0213 (10)	0.0182 (8)	−0.0034 (8)	−0.0007 (7)	−0.0036 (7)
C8	0.0196 (10)	0.0210 (10)	0.0210 (9)	−0.0017 (8)	0.0007 (7)	−0.0046 (7)
C9	0.0274 (11)	0.0256 (11)	0.0248 (9)	−0.0066 (9)	0.0001 (8)	−0.0063 (8)
C10	0.0268 (11)	0.0261 (11)	0.0388 (11)	−0.0105 (9)	−0.0085 (8)	−0.0056 (9)
C11	0.0303 (11)	0.0261 (11)	0.0237 (9)	−0.0086 (9)	−0.0048 (8)	−0.0070 (8)
C12	0.0197 (10)	0.0195 (10)	0.0180 (8)	−0.0042 (8)	0.0010 (7)	−0.0057 (7)
C13	0.0210 (10)	0.0241 (11)	0.0265 (9)	0.0005 (8)	−0.0025 (8)	−0.0106 (8)
C14	0.0298 (11)	0.0260 (11)	0.0246 (9)	−0.0039 (9)	−0.0089 (8)	−0.0062 (8)
C15	0.0329 (12)	0.0238 (10)	0.0190 (9)	−0.0065 (9)	−0.0011 (8)	−0.0070 (8)
C16	0.0251 (11)	0.0239 (10)	0.0247 (9)	−0.0023 (8)	0.0011 (8)	−0.0079 (8)
C17	0.0222 (10)	0.0216 (10)	0.0234 (9)	−0.0027 (8)	−0.0040 (8)	−0.0057 (8)

### Geometric parameters (Å, °)

**Table:**

O1—C3	1.3848 (17)	C8—C9	1.491 (2)
O1—C10	1.4367 (17)	C9—H9A	0.9800
N1—C8	1.3891 (18)	C9—H9B	0.9800
N1—C1	1.3984 (19)	C9—H9C	0.9800
N1—C12	1.4428 (18)	C10—H10A	0.9800
N2—C11	1.1491 (17)	C10—H10B	0.9800
C1—C2	1.392 (2)	C10—H10C	0.9800
C1—C6	1.4074 (18)	C12—C17	1.379 (2)
C2—C3	1.384 (2)	C12—C13	1.388 (2)
C2—H2	0.9500	C13—C14	1.388 (2)
C3—C4	1.4028 (19)	C13—H13	0.9500
C4—C5	1.386 (2)	C14—C15	1.383 (2)
C4—H4	0.9500	C14—H14	0.9500
C5—C6	1.394 (2)	C15—C16	1.382 (2)
C5—H5	0.9500	C15—H15	0.9500
C6—C7	1.445 (2)	C16—C17	1.3908 (19)
C7—C8	1.375 (2)	C16—H16	0.9500
C7—C11	1.424 (2)	C17—H17	0.9500
			
C3—O1—C10	118.09 (12)	H9A—C9—H9B	109.5
C8—N1—C1	109.28 (12)	C8—C9—H9C	109.5
C8—N1—C12	125.76 (13)	H9A—C9—H9C	109.5
C1—N1—C12	124.74 (12)	H9B—C9—H9C	109.5
C2—C1—N1	129.25 (13)	O1—C10—H10A	109.5
C2—C1—C6	122.54 (14)	O1—C10—H10B	109.5
N1—C1—C6	108.17 (13)	H10A—C10—H10B	109.5
C3—C2—C1	117.25 (13)	O1—C10—H10C	109.5
C3—C2—H2	121.4	H10A—C10—H10C	109.5
C1—C2—H2	121.4	H10B—C10—H10C	109.5
C2—C3—O1	115.18 (13)	N2—C11—C7	178.9 (2)
C2—C3—C4	121.71 (14)	C17—C12—C13	121.16 (15)
O1—C3—C4	123.10 (14)	C17—C12—N1	119.28 (15)
C5—C4—C3	119.96 (15)	C13—C12—N1	119.56 (14)
C5—C4—H4	120.0	C14—C13—C12	119.08 (15)
C3—C4—H4	120.0	C14—C13—H13	120.5
C4—C5—C6	119.97 (14)	C12—C13—H13	120.5
C4—C5—H5	120.0	C15—C14—C13	119.90 (16)
C6—C5—H5	120.0	C15—C14—H14	120.0
C5—C6—C1	118.55 (14)	C13—C14—H14	120.0
C5—C6—C7	135.72 (14)	C16—C15—C14	120.69 (15)
C1—C6—C7	105.69 (13)	C16—C15—H15	119.7
C8—C7—C11	123.12 (16)	C14—C15—H15	119.7
C8—C7—C6	108.95 (13)	C15—C16—C17	119.69 (16)
C11—C7—C6	127.90 (15)	C15—C16—H16	120.2
C7—C8—N1	107.91 (14)	C17—C16—H16	120.2
C7—C8—C9	129.08 (14)	C12—C17—C16	119.42 (16)
N1—C8—C9	123.01 (13)	C12—C17—H17	120.3
C8—C9—H9A	109.5	C16—C17—H17	120.3
C8—C9—H9B	109.5		
			
C8—N1—C1—C2	−177.94 (16)	C11—C7—C8—N1	177.40 (15)
C12—N1—C1—C2	7.2 (3)	C6—C7—C8—N1	−0.93 (19)
C8—N1—C1—C6	−0.32 (18)	C11—C7—C8—C9	−3.4 (3)
C12—N1—C1—C6	−175.17 (14)	C6—C7—C8—C9	178.30 (17)
N1—C1—C2—C3	178.75 (16)	C1—N1—C8—C7	0.78 (19)
C6—C1—C2—C3	1.4 (2)	C12—N1—C8—C7	175.56 (15)
C1—C2—C3—O1	178.75 (14)	C1—N1—C8—C9	−178.50 (16)
C1—C2—C3—C4	−1.5 (2)	C12—N1—C8—C9	−3.7 (3)
C10—O1—C3—C2	−173.49 (15)	C8—C7—C11—N2	−83 (10)
C10—O1—C3—C4	6.8 (2)	C6—C7—C11—N2	95 (10)
C2—C3—C4—C5	0.6 (3)	C8—N1—C12—C17	−112.79 (18)
O1—C3—C4—C5	−179.64 (16)	C1—N1—C12—C17	61.2 (2)
C3—C4—C5—C6	0.4 (3)	C8—N1—C12—C13	67.1 (2)
C4—C5—C6—C1	−0.4 (3)	C1—N1—C12—C13	−118.94 (17)
C4—C5—C6—C7	−177.77 (18)	C17—C12—C13—C14	2.7 (2)
C2—C1—C6—C5	−0.5 (2)	N1—C12—C13—C14	−177.16 (14)
N1—C1—C6—C5	−178.30 (15)	C12—C13—C14—C15	−2.4 (2)
C2—C1—C6—C7	177.58 (15)	C13—C14—C15—C16	0.4 (2)
N1—C1—C6—C7	−0.24 (18)	C14—C15—C16—C17	1.5 (2)
C5—C6—C7—C8	178.28 (19)	C13—C12—C17—C16	−0.8 (2)
C1—C6—C7—C8	0.72 (19)	N1—C12—C17—C16	179.00 (14)
C5—C6—C7—C11	0.1 (3)	C15—C16—C17—C12	−1.3 (2)
C1—C6—C7—C11	−177.50 (17)		

### Hydrogen-bond geometry (Å, °)

**Table:**

Cg1, Cg2 and Cg3 are the centroids of the N1/C1/C6–C8, C1–C6 and C12–C17 rings, respectively.

**Table:**

*D*—H···*A*	*D*—H	H···*A*	*D*···*A*	*D*—H···*A*
C9—H9B···Cg2^i^	0.98	2.78	3.701 (2)	156
C10—H10B···Cg3^ii^	0.98	2.65	3.516 (2)	148
C10—H10C···Cg1^iii^	0.98	2.73	3.509 (2)	137

Symmetry codes: (i) *x*−1, *y*, *z*; (ii) −*x*+2, −*y*+1, −*z*; (iii) *x*+1, *y*, *z*.
